# Investigation of Mitochondrial Dysfunction by Sequential Microplate-Based Respiration Measurements from Intact and Permeabilized Neurons

**DOI:** 10.1371/journal.pone.0034465

**Published:** 2012-04-04

**Authors:** Pascaline Clerc, Brian M. Polster

**Affiliations:** Department of Anesthesiology and Center for Shock, Trauma and Anesthesiology Research, University of Maryland School of Medicine, Baltimore, Maryland, United States of America; Université Joseph Fourier, France

## Abstract

Mitochondrial dysfunction is a component of many neurodegenerative conditions. Measurement of oxygen consumption from intact neurons enables evaluation of mitochondrial bioenergetics under conditions that are more physiologically realistic compared to isolated mitochondria. However, mechanistic analysis of mitochondrial function in cells is complicated by changing energy demands and lack of substrate control. Here we describe a technique for sequentially measuring respiration from intact and saponin-permeabilized cortical neurons on single microplates. This technique allows control of substrates to individual electron transport chain complexes following permeabilization, as well as side-by-side comparisons to intact cells. To illustrate the utility of the technique, we demonstrate that inhibition of respiration by the drug KB-R7943 in intact neurons is relieved by delivery of the complex II substrate succinate, but not by complex I substrates, via acute saponin permeabilization. In contrast, methyl succinate, a putative cell permeable complex II substrate, failed to rescue respiration in intact neurons and was a poor complex II substrate in permeabilized cells. Sequential measurements of intact and permeabilized cell respiration should be particularly useful for evaluating indirect mitochondrial toxicity due to drugs or cellular signaling events which cannot be readily studied using isolated mitochondria.

## Introduction

Mitochondria generate ATP via oxidative phosphorylation, produce and detoxify reactive oxygen species production, participate in intracellular calcium regulation, and control the onset of intrinsic apoptosis [1], [2]. Extensive evidence implicates mitochondrial dysfunction and associated oxidative stress in aging and neurodegenerative diseases such as Parkinson's disease, Alzheimer's disease, Huntington's disease, amyotrophic lateral sclerosis, and stroke [3], [4]. Although defects in specific electron transport chain complexes, e.g. complex I in Parkinson's disease and complex IV in Alzheimer's disease, are thought to contribute to the pathogenesis of these disorders, the complex interplay among calcium changes, oxidative stress, alterations in mitochondrial fission/fusion, and bioenergetics is incompletely understood.

The study of individual electron transport chain components in isolated mitochondria using specific substrates and inhibitors has been successful in elucidating many facets of mitochondrial biology, including bioenergetic dysfunction in neurodisease. However, it is increasingly appreciated that some events that impact mitochondrial function, such as shape changes, interaction with other organelles, and mitophagy—the removal of damaged mitochondria—cannot be fully modeled once mitochondria are separated from the cell. An alternative to mitochondrial isolation is cell permeabilization. Saponin and digitonin are steroid glycosides which porate membranes by extracting cholesterol [5]. Because the plasma membrane has a greater molar ratio of cholesterol to phospholipid than intracellular membranes, carefully titrated digitonin or saponin permeabilizes the cell membrane without compromising the integrity of organelles or the cytoskeleton [5], [6]. This technique enables the mechanistic study of mitochondria without isolation.

Traditionally the measurement of mitochondrial oxygen consumption from permeabilized cells required in excess of two million cells, detachment of cells from a substrate, and resuspension in an artificial intracellular-like milieu that includes high (∼125 mM) potassium and little to no calcium or sodium [7]. These constraints limited the utility of the technique for cultured neurons which are typically obtained from rodent embryos in limited numbers and degenerate following detachment and resuspension. Additionally, the different assay conditions typically used for intact and permeabilized cells, particularly millimolar extracellular calcium which causes mitochondrial calcium overload if cells are permeabilized, have not allowed successive measurements from intact and permeabilized cells.

This study tested the hypothesis that the Extracellular Flux Analyzer (Seahorse Bioscience, North Billerica, MA), a microplate-based respirometer designed to measure O_2_ consumption rate (OCR) from adherent cells, could be adapted for OCR measurements from permeabilized neurons and used to identify electron transport chain deficits. Because bioenergetic function of isolated mitochondria is preserved in a sodium-based assay medium [8], [9], we hypothesized that sequential measurements could be made from intact and acutely permeabilized cells if the calcium concentration is controlled following permeabilization. The drug KB-R7943 inhibits complex I in isolated brain mitochondria and blocks sodium-calcium exchangers (NCXs) and NMDA-subtype glutamate receptors at the neuronal plasma membrane [10], [11]. To investigate the feasibility and utility of sequential permeabilization, we first treated intact neurons with KB-R7943, allowing it to act on both mitochondrial and plasma membrane drug targets. The cell-impermeable complex II substrate succinate was then delivered by acute permeabilization or cell permeable methyl succinate was added to intact cells to test whether inhibition of respiration by KB-R7943 in cells is primarily due to impairment of complex I.

ADP-stimulated mitochondrial respiration and coupling were equally preserved in sodium or potassium-based assay medium when calcium was controlled by chelation with EGTA. KB-R7943-inhibited respiration was rescued by bypassing complex I with succinate but not by methyl succinate, establishing proof-of-principle that sequential permeabilization can be used to investigate complex I deficiencies in neurons.

## Methods

### Materials

KB-R7943 was purchased from Tocris Bioscience (Ellisville, MO). Cell culture supplies were from Invitrogen (Grand Island, NY). Protease Inhibitor Cocktail Set III was from EMD Biosciences (San Diego, CA). All other reagents, including purified horse heart cytochrome *c*, were from Sigma-Aldrich (St. Louis, MO). Pyruvate and saponin were made fresh from powder and pH-adjusted for each individual experiment. Other reagents were diluted from concentrated pH-adjusted stocks stored at −20°C.

### Preparation of primary neurons

#### Ethics Statement

All procedures were approved by the University of Maryland Institutional Animal Care and Use Committee (IACUC protocol # 1109008) and were in accordance with the NIH Guide for the Care and Use of Laboratory Animals. Primary cortical neurons were prepared from E18 rat cortices by trypsin dissociation as described [12], [13]. Cells were seeded in V7 microplates (Seahorse Bioscience) at a density of 80,000 cells/well (0.32 cm^2^) in 100 µl of Neurobasal medium containing B27 supplement (2%), L-glutaMAX (0.5 mM), fetal bovine serum (10%), penicillin (100 IU/ml) and streptomycin (100 µg/ml). After two hours when the majority of cells had attached, medium was aspirated and replaced with 0.5 ml of the same culture medium but lacking fetal bovine serum. At 4 days in vitro (DIV), cytosine arabinofuranoside (5 µM) was added to inhibit glial proliferation. On DIV 6, 0.2 ml of fresh Neurobasal medium containing supplements was added to each well. Neurons were maintained at 37°C and physiologically relevant O_2_ in an oxygen-regulated incubator with a humidified atmosphere of 92% N_2_/5% CO_2_/3% O_2_ as previously described [14]. Cells were used for experiments at 11–15 DIV.

### Measurement of O_2_ consumption by XF24 microplate-based respirometry

O_2_ consumption measurements from intact and permeabilized neurons were made using an XF24 Extracellular Flux Analyzer (Seahorse Bioscience). All but 50 µl of Neurobasal/B27 culture medium was removed from each well and cells were washed twice in 0.5 ml of aCSF assay medium that consisted of (in mM) 120 NaCl, 3.5 KCl, 1.3 CaCl_2_, 0.4 KH_2_PO_4_, 1 MgCl_2_, 20 HEPES, and 15 glucose, pH 7.2. Medium was additionally supplemented with 4 mg/ml fatty acid free bovine serum album (BSA, Sigma-Aldrich, catalogue No. A6003). A few experiments with intact cells were performed in aCSF containing a reduced concentration of HEPES (5 mM). Cells were incubated in 0.675 ml aCSF at 37°C in a CO_2_-free incubator for one hour prior to measurements. For experiments comparing KCl-based assay medium to aCSF, aCSF medium was replaced by KCl medium at the conclusion of this one hour incubation period and just prior to the start of measurements. KCl medium consisted of 125 mM KCl, 2 mM K_2_HPO_4_, 1 mM MgCl_2_, 20 mM HEPES, 4 mg/ml BSA and 15 mM glucose, pH 7.0.

Following the equilibration period, cells were loaded into the XF24 and further equilibrated for 15 min by three 3 min mix, 2 min wait cycles prior to the first measurement. XF assays consisted of cycles of 0.5 min mix, 0.5 min wait, and 3 min measurement for most experiments and were performed at 37°C as described [15], [16]. A 3 min mix, 2 min wait, 2 min measurement cycle was used in a few cases. Compounds of interest prepared in assay medium (75 µl) were preloaded into reagent delivery chambers *a*, *b*, *c*, and *d* at 10×, 11×, 12×, and 13× the final working concentration, respectively, and injected sequentially as described in figure legends.

Saponin (25 µg/ml) was co-injected with 3.6 mM K_2_HPO_4_, 1 mM ADP, 5 mM EGTA, and mitochondrial substrate(s) as indicated to initiate permeabilization and ADP-stimulated respiration in aCSF assay medium. Because binding of calcium to EGTA causes acidification [17], EGTA was added from a 250 mM stock in aCSF that was pH-adjusted to 7.4. Assay medium pH was reduced to 7.0 following injection of the saponin/EGTA mixture, compatible with the measurement of mitochondrial O_2_ consumption in permeabilized cells. For assays conducted in KCl medium, saponin (25 µg/ml) was co-injected with 2 mM K_2_HPO_4_, 1 mM ADP, and mitochondrial substrate(s) as indicated. A final concentration of 4 mM K_2_HPO_4_ was determined by titration to yield maximal ADP-stimulated respiration (data not shown). Substrate combinations for complex I-linked respiration consisted of 5 mM pyruvate/5 mM malate, 5 mM glutamate/5 mM malate, or 3 mM glutamate/1 mM malate, as described in figure legends. Succinate (5 mM or 3 mM) or methyl succinate (5 mM) in combination with rotenone (0.5 µM) or glutamate (3 mM) was used to assay complex II-dependent respiration as described below. The ATP synthase inhibitor oligomycin (0.3 mg/ml) was used to measure OCR in the absence of oxidative phosphorylation, the protonophore carbonyl cyanide 4-(trifluoromethoxy) phenylhydrazone (FCCP, 2 µM) was added to measure uncoupled respiration, and the complex III inhibitor antimycin A (1 µM) was used to inhibit O_2_ consumption by the mitochondrial electron transport chain.

### Calculation of respiratory control ratio (RCR)

We used the method of Sauerbeck et al. to calculate RCR using absolute oxygen consumption rates (in pmol O_2_/min/80,000 plated cells) measured by the XF24 [18]. This method uses the point-by-point O_2_ consumption rates calculated with the AKOS algorithm over each measurement period [16], [18]. The peak point-by-point ADP rate was used. Oligomycin and antimycin A OCRs were calculated as the average of the last three point-by-point measurements when maximum effect was attained. OCR following antimycin A addition was subtracted to exclude non-mitochondrial respiration [19]. Additionally, because OCRs following oligomycin or antimycin A were often close to background, we did not use average background correction when calculating RCR to minimize the influence of small fluctuations in individual temperature control wells. The subtraction of OCR measured in the presence of antimycin A in individual wells excludes not only non-mitochondrial cellular O_2_ consumption but any apparent background O_2_ consumption due to properties of the O_2_-sensitive fluorophore. RCR was calculated as (ADP OCR-antimycin A OCR)/(oligomycin OCR-antimycin A OCR).

### Data analysis and statistics

**Figure 1:**
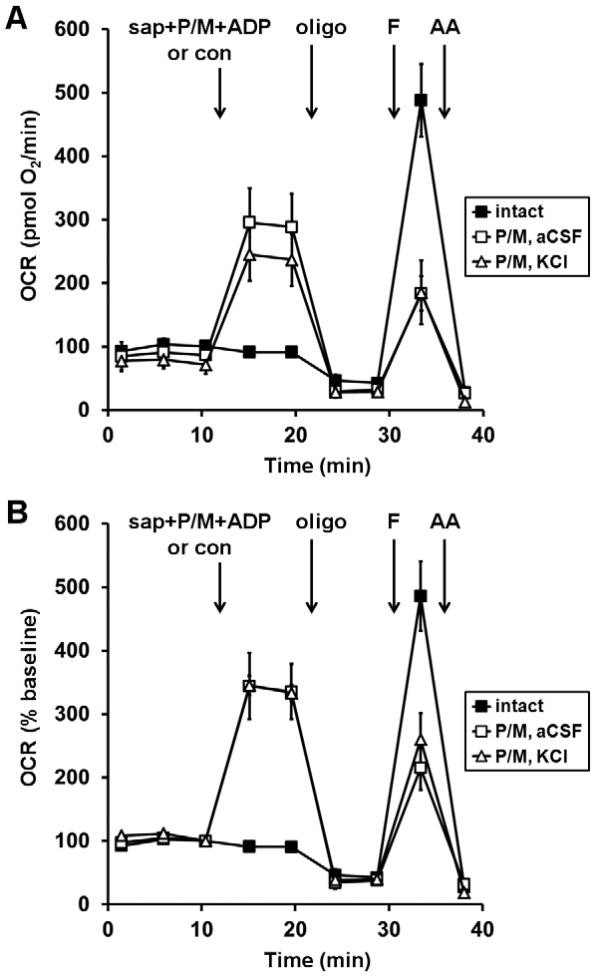
Comparison of saponin-permeabilized neuronal respiration in aCSF vs. KCl-based medium. (**A**) Primary rat cortical neurons were control-treated in aCSF (con, filled squares) or permeabilized by saponin (sap, 25 µg/ml) in aCSF (open squares) or KCl-based assay medium (open triangles) after three baseline O_2_ consumption rate (OCR) measurements (first arrow). Respiration was stimulated by co-injection of ADP (1 mM) in the presence of the complex I substrates pyruvate and malate (P/M, 5 mM each). K_2_HPO_4_ was also co-injected with saponin to obtain a final concentration of 4 mM in each assay medium (see Methods) and EGTA (5 mM) was included for cells assayed in aCSF. Oligomycin (oligo, 0.3 µg/ml), FCCP, (F, 2 µM) and antimycin A (AA, 1 µM) were subsequently added as indicated (arrows). Pyruvate (10 mM) was included with FCCP for intact cells (filled squares) here and in subsequent experiments to insure that substrate supply was not rate-limiting for uncoupled respiration. OCRs were measured in triplicate (mean ± SD). (**B**) OCRs in (**A**) baseline-normalized to the point prior to sap or con addition.

For comparing OCR after compound exposure to OCR pre-exposure, the absolute cell number is irrelevant since the same population of cells is compared. Therefore, to reduce variability due to slight differences in plating and viability during the 11–15 DIV culture period, most results are depicted as normalized OCR (% baseline rate) for each individual population of cells, as previously described [15], [20]. Both absolute and baseline-normalized OCRs are shown in Fig. 1. The medians of 3–5 technical replicates from four independent experiments were averaged to determine state 3 (ADP-stimulated), state 4 (oligomycin-inhibited), and uncoupled OCRs (Table 1). One-way analysis of variance with repeated measures was employed to evaluate statistical significance, with p<0.05 considered significant. Tukey's post-hoc analysis was used to compare individual groups. Statistical analyses were carried out using SigmaStat 3.5 (Systat Software, Inc., San Jose, CA).

**Table 1:** Respiration rates and respiratory control ratios for permeabilized neurons oxidizing various substrates.

Substrate	Initial	State 3	State 4	uncoupled	RCR
P/M	63±6	280±48	25±4	200±47*	12.1±2.7
S/R	70±14	322±59	52±13*	170±26*	6.5±0.6
G/M	66±8	280±37	37±8	100±16*,#	9.2±2.6
Intact	69±6	N/A	16±6	424±60	N/A

Initial, state 3, state 4, and uncoupled O_2_ consumption rates (OCRs) are expressed in pmol O_2_/min/80,000 plated cells. The initial OCRs are the rates measured just prior to permeabilization or control injection (intact). The state 4 rates were measured in the presence of oligomycin. The respiratory control ratio (RCR) is the ratio of the state 3 rate to the state 4 rate, calculated on an individual well basis prior to averaging. Data are mean ± SE, n = 4 experiments of 3–5 wells per treatment. One well (P/M) was excluded from analysis because the antimycin OCR was higher than the oligomycin OCR.

* indicates a significant difference compared to intact cells (p<0.05). # indicates a significant difference compared to pyruvate/malate (p<0.05).

Data in figures are presented as mean ± SD from individual experiments and are representative of at least three similar experiments.

## Results

Previous studies established that treatment with 25–50 µg/ml saponin permeabilizes neuronal plasma membrane without compromising mitochondrial structure [7], [21], [22]. We measured O_2_ consumption by intact neurons in either a typical, nominally calcium-free KCl-based intracellular assay medium or a standard artificial cerebrospinal fluid (aCSF) solution and evaluated the response to the co-injection of saponin (25 µg/ml), mitochondrial complex I-linked substrates, ADP, and excess phosphate. EGTA (5 mM) was additionally co-injected with saponin for aCSF-incubated neurons to yield ∼107 nM free calcium, approximating free calcium in the cytoplasm (calculated using Theo Schoenmakers' Chelator software, http://www.stanford.edu/~cpatton/CaMgATPEGTA-TS-Plot.htm) [17]. Basal or ADP-stimulated state 3 (phosphorylating) respiration did not differ between aCSF and KCl medium (Fig. 1A). Addition of saponin and substrate increased OCRs by >300% compared to non-permeabilized neurons injected with control solution (Fig. 1B). As expected, injection of saponin to aCSF-incubated neurons without EGTA caused a rapid and complete loss of respiration (data not shown), consistent with calcium-mediated mitochondrial damage.

The ATP synthase inhibitor oligomycin was added after ADP to evaluate mitochondrial coupling. When H+ flux through the ATP synthase is inhibited, phosphorylating respiration stops and residual O_2_ consumption is primarily due to proton leak across the mitochondrial inner membrane. Oligomycin decreased OCR to the same level in intact and permeabilized neurons assayed side-by-side on the same microplate (Fig. 1). This finding indicates that mitochondria within permeabilized neurons remain coupled in both sodium and potassium-based assay medium. Next, we injected the uncoupler FCCP to examine maximal respiratory capacity. FCCP dissipates the H+ gradient across the mitochondrial inner membrane, uncoupling electron transport from oxidative phosphorylation. In the presence of FCCP, OCR increases to the maximum extent supported by the electron transport chain and substrate supply. FCCP stimulated respiration to a greater degree in intact neurons than in neurons permeabilized in either KCl or aCSF (Fig. 1). However, the ability of FCCP to stimulate respiration in aCSF medium was similar to that observed in KCl medium. Because bioenergetic function was relatively well-preserved in neurons permeabilized in aCSF, subsequent optimization was performed using this assay medium to allow for sequential measurements from intact and permeabilized cells.

**Figure 2:**
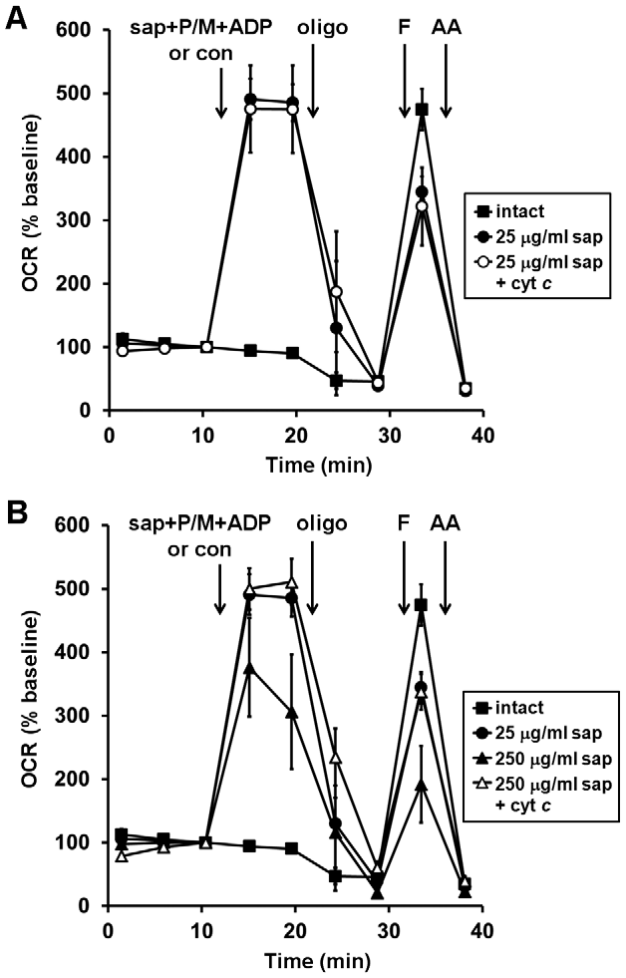
Optimized plasma membrane permeabilization by saponin does not compromise mitochondrial outer membrane integrity. (**A**) Primary rat cortical neurons were control-treated (con, filled squares) or permeabilized by saponin (sap, 25 µg/ml) plus EGTA (5 mM) in aCSF medium in the absence (filled circles) or presence (open circles) of purified cytochrome *c* (100 µM) after three baseline O_2_ consumption rate (OCR) measurements (first arrow). Pyruvate and malate (P/M, 5 mM each), ADP (1 mM), and excess K_2_PHO_4_ (3.6 mM for 4 mM final) were co-injected with saponin to measure complex I-dependent ADP-stimulated respiration. Subsequently, oligomycin (oligo, 0.3 µg/ml), FCCP, (F, 2 µM) and antimycin A (AA, 1 µM) were added as indicated (arrows). Pyruvate (10 mM) was included with FCCP for intact cells (filled squares). (**B**) Neurons were permeabilized as described in (**A**) using 250 µg/ml saponin in the absence (filled triangles) or presence (open triangles) of purified cytochrome *c* (100 µM). Control-treated neurons (filled squares) and neurons permeabilized by 25 µg/ml saponin (filled circles) are duplicated from (**A**) for comparison. OCRs in (**A**) and (**B**) are mean ± SD in quadruplicate, normalized to the third measurement point and expressed as % baseline OCR. All treatments in (**A**) and (**B**) were assayed in parallel on an individual 24-well plate.

Excessive concentrations of saponin can permeabilize the mitochondrial outer membrane as well as the plasma membrane. Mitochondrial outer membrane permeabilization leads to loss of cytochrome *c*, the electron carrier between complex III and complex IV, which causes a reduction in respiratory capacity [23]. To confirm that mitochondrial outer membrane integrity was not compromised in permeabilized neurons, leading to a limitation in ADP or FCCP-stimulated OCR, exogenous purified cytochrome *c* was co-injected with saponin. Cytochrome *c* had no effect on either state 3 or uncoupled respiration in neurons permeabilized by 25 µg/ml saponin (Fig. 2A). However, injection of a ten-fold higher saponin concentration reduced both ADP-stimulated and uncoupled respiration and these reductions were completely prevented by exogenous cytochrome *c* (Fig. 2B). These results indicate that 250 µg/ml saponin, but not 25 µg/ml saponin, results in partial mitochondrial outer membrane permeabilization that limits OCR due to cytochrome *c* release. In addition, they demonstrate that cytochrome *c* loss was not responsible for the reduced FCCP response in neurons permeabilized by 25 µg/ml saponin compared to intact cells.

**Figure 3:**
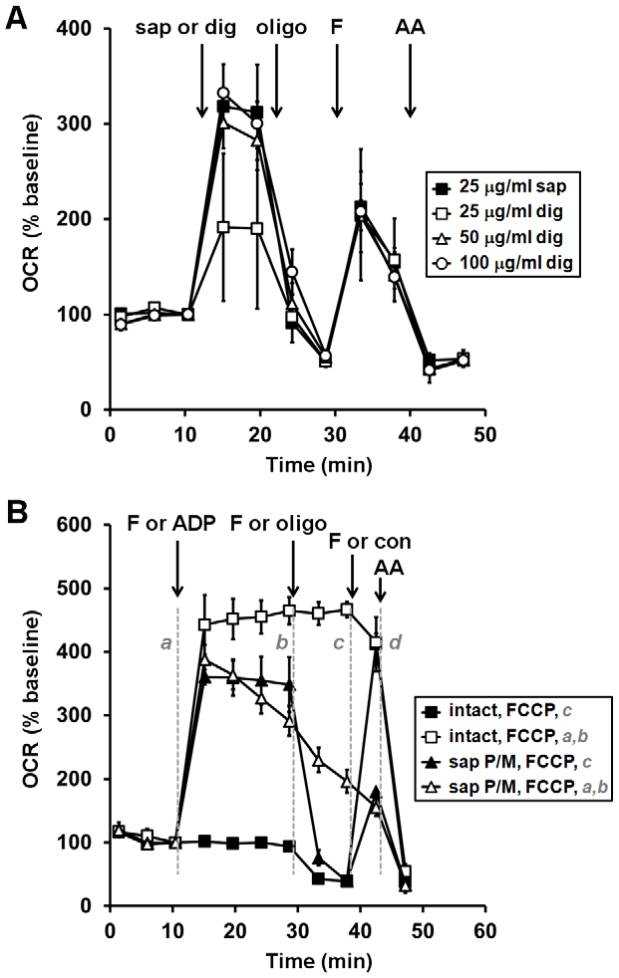
Effect of the permeabilizing agent and time on FCCP-stimulated respiration. (**A**) Primary rat cortical neurons were permeabilized by saponin (sap, 25 µg/ml, filled squares) or digitonin (25 µg/ml, open squares, 50 µg/ml, open triangles, 100 µg/ml, open circles) plus EGTA (5 mM) in aCSF medium after three baseline O_2_ consumption rate (OCR) measurements (first arrow). Pyruvate and malate (P/M, 5 mM each), ADP (1 mM), and excess K_2_PHO_4_ (3.6 mM for 4 mM final) were co-injected with saponin to measure complex I-dependent ADP-stimulated respiration. Oligomycin (oligo, 0.3 µg/ml), FCCP, (F, 2 µM) and antimycin A (AA, 1 µM) were added as indicated (arrows). (**B**) OCRs were measured and at the injection marked *a*, neurons were control-treated in the absence (filled squares) or presence of FCCP plus pyruvate (2 µM and 10 mM, respectively, open squares) or permeabilized using saponin (triangles). Complex I-linked respiration (P/M) in permeabilized neurons was stimulated by ADP (1 mM, filled triangles) or FCCP (F, 2 µM, open triangles). Intact (open squares) and permeabilized (open triangles) cells treated with FCCP received a second FCCP injection (1 µM) at *b* to insure respiration was maximally uncoupled, followed by a control injection at *c* and finally antimycin A (AA, 1 µM) at *d*. Control-treated intact cells (filled squares) and ADP-treated permeabilized cells (filled triangles) received injections of oligo, FCCP, (F, 2 µM) and AA in ports *b*, *c*, and *d*, respectively, with pyruvate (10 mM) included with FCCP for intact cells. OCRs in (**A**) and (**B**) are mean ± SD in quadruplicate, normalized to the third measurement point and expressed as % baseline OCR.

Some studies used digitonin in place of saponin to achieve selective plasma membrane permeabilization [6], [24]. We tested a range of saponin and digitonin concentrations and found that although digitonin effectively permeabilized neurons at ≥50 µg/ml, we were unable to increase ADP or FCCP-stimulated OCRs beyond those achieved by 25 µg/ml saponin (Fig. 3A and data not shown). In the experiments described in Figs. 1 and 2, ADP was co-injected with saponin and substrate but FCCP was injected after nearly 20 minutes. To test the hypothesis that the impaired FCCP response in permeabilized cells relative to intact cells was time-dependent and/or due to the prior injection of oligomycin, we either injected FCCP simultaneous to saponin permeabilization and measured respiration continuously, or injected FCCP after oligomycin addition nearly 30 minutes later. FCCP stimulated respiration to the same level as ADP, and nearly to the level of FCCP-treated intact cells, when injected simultaneous to the saponin addition (Fig. 3B, injection *a*, open triangles). However, the FCCP response was greatly reduced when FCCP was added >20 minutes after the saponin injection (injection *c*, filled triangles). ADP-stimulated respiration was stable over a 20 minute period (injection *a*, filled triangles). However, FCCP-stimulated respiration declined progressively in permeabilized (open triangles) but not in intact cells (open squares). Injection of additional FCCP (Fig. 3B) or additional mitochondrial substrate (data not shown) failed to improve uncoupled respiration.

The ratio of ADP-stimulated, state 3 respiration to state 4, non-phosphorylating respiration is referred to as the respiratory control ratio (RCR). The greater the RCR, the better coupled the mitochondria, and the greater the efficiency of ATP synthesis. We estimated RCRs in permeabilized neurons using the substrates pyruvate/malate or glutamate/malate (complex I-linked respiration), or succinate in the presence of the complex I inhibitor rotenone (complex II-linked respiration). RCRs for permeabilized neurons oxidizing complex I or complex II substrates were comparable to or superior than previous estimates using isolated mitochondria from primary cortical neurons or rat brain (Table 1) [18], [25]–[27]. State 4 respiration was significantly higher when the complex II substrate succinate was used compared to the utilization of complex I substrates, consistent with results obtained using mitochondria isolated from cortical neurons [25]. FCCP-stimulated OCR (measured after oligomycin addition) was significantly lower with any of the substrate combinations compared to the FCCP rate in intact cells. FCCP-stimulated OCR was also significantly lower for the substrates glutamate/malate compared to pyruvate/malate.

**Figure 4:**
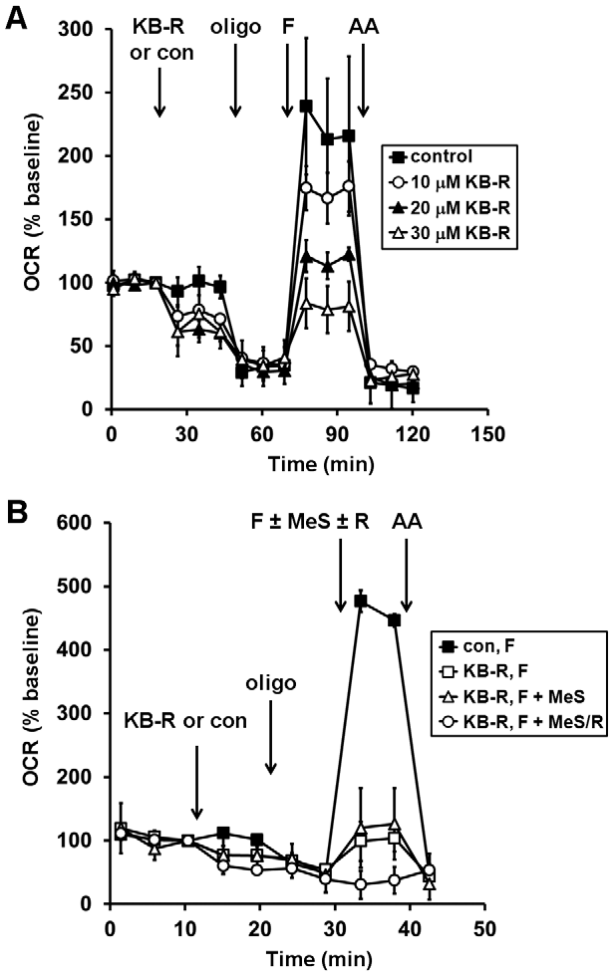
Methyl succinate fails to rescue KB-R7943-inhibited respiration in intact neurons. (**A**) Primary rat cortical neurons were control-treated (con, filled squares) or treated with KB-7943 (K-BR, 10 µM, open circles, 20 µM, filled triangles, or 30 µM, open triangles) after three baseline O_2_ consumption rate (OCR) measurements (first arrow). Subsequently, oligomycin (oligo, 0.3 µg/ml), FCCP plus pyruvate (F, 2 µM and 10 mM, respectively), and antimycin A (AA, 1 µM) were added as indicated (arrows). (**B**) Neurons were control-treated (con, filled squares) or treated with KB-7943 (30 µM, all other groups) as in (**A**). Other additions were as (A) except the FCCP+pyruvate injection after KB-R7943 addition also contained methyl succinate (MeS, 10 mM, open triangles), methyl succinate plus rotenone (10 mM/0.5 µM, MeS/R, open circles), or no additional substrate (open squares). OCRs in (**A**) and (**B**) are mean ± SD in triplicate, normalized to the third measurement point and expressed as % baseline OCR.

**Figure 5:**
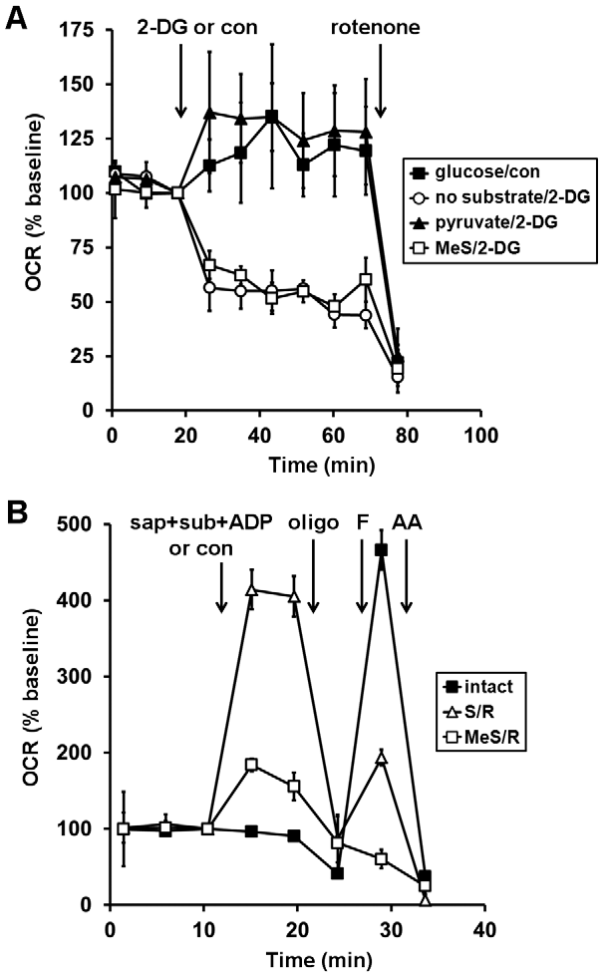
Methyl succinate is a poor substrate for complex II. (**A**) Primary rat cortical neurons were incubated in aCSF in the presence of glucose (15 mM, filled squares), no substrate (open circles), pyruvate (10 mM, filled triangles), or methyl succinate (10 mM, open squares). Cells were control-treated (con, filled squares) or treated with 2-deoxyglucose (2-DG, 2 mM, all other groups) after three baseline O_2_ consumption rate (OCR) measurements (first arrow). Rotenone (0.5 µM) was subsequently added (second arrow) to inhibit complex I. (**B**) Neurons were control-treated (con, filled squares) or permeabilized by saponin (sap, 25 µg/ml) plus EGTA (5 mM) in aCSF medium after three baseline O_2_ consumption rate (OCR) measurements (first arrow). Succinate/rotenone (S/R, 5 mM and 0.5 mM respectively) or methyl succinate/rotenone (MeS/R, 5 mM and 0.5 mM respectively) as substrate (sub), ADP (1 mM), and excess K_2_PHO_4_ (3.6 mM for 4 mM final) were co-injected with saponin to measure complex II-dependent ADP-stimulated respiration. Subsequently, oligomycin (oligo, 0.3 µg/ml), FCCP, (F, 2 µM) and antimycin A (AA, 1 µM) were added as indicated (arrows). Pyruvate (10 mM) was included with FCCP for intact cells (filled squares) to insure that substrate supply was not rate-limiting for uncoupled respiration. OCRs in (**A**) and (**B**) are mean ± SD in triplicate, normalized to the third measurement point and expressed as % baseline OCR.

Having demonstrated that coupled respiration can be measured using either complex I- or complex II-linked substrates in neurons permeabilized in aCSF, we used this technique to investigate the mechanism of neuronal respiratory inhibition by KB-R7943, a drug with multiple targets. First, we confirmed the ability of KB-R7943 to inhibit O_2_ consumption in intact neurons and investigated whether methyl succinate, a putative cell permeable complex II-linked substrate, could relieve respiratory inhibition. Treatment with KB-R7943 (10–30 µM) led to an immediate 25–40% inhibition of basal OCR and a dose-dependent attenuation of respiratory capacity in primary cortical neurons (Fig. 4A), consistent with effects reported in hippocampal neurons [10]. Although KB-R7943 was shown to inhibit complex I-linked but not complex II-linked respiration in isolated brain mitochondria [10], surprisingly KB-R7943-inhibited respiration in intact neurons was not rescued by methyl succinate (Fig. 4B). To test whether methyl succinate could support respiration in neurons in the absence of other substrates, 2-deoxyglucose (2-DG), a competitive inhibitor of glycolysis, was added to glucose-deprived neurons. Neurons were able to support basal OCR for a short time in the absence of exogenous substrate (Fig. 5A, open circles). However, 2-DG (2 mM) inhibited OCR by ∼50% in the absence of glucose (open circles) and respiration was further reduced to ∼10% of the basal rate by the addition of the complex I inhibitor rotenone (Fig. 5A). Pyruvate, a cell permeable complex I substrate (filled triangles), but not methyl succinate (open squares) occluded the ability of 2-DG to inhibit respiration (Fig. 5A). Residual respiration in the presence of methyl succinate and 2-DG was rotenone sensitive, indicating that methyl succinate was not able to support complex II-dependent respiration in intact neurons in the absence of glycolysis (Fig. 5A).

To test whether the inability of methyl succinate to support complex II-dependent respiration was due to its inability to penetrate cells, we compared state 3, state 4, and uncoupled respiration in neurons permeabilized by saponin in the presence of rotenone (0.5 mM) and an equimolar concentration of methyl succinate or succinate (5 mM). ADP and FCCP stimulated OCRs were greatly reduced when methyl succinate rather than succinate was supplied as the lone exogenous substrate (Fig. 5B). These results indicate that methyl succinate is a poor complex II substrate, irrespective of its ability to permeate cells.

**Figure 6:**
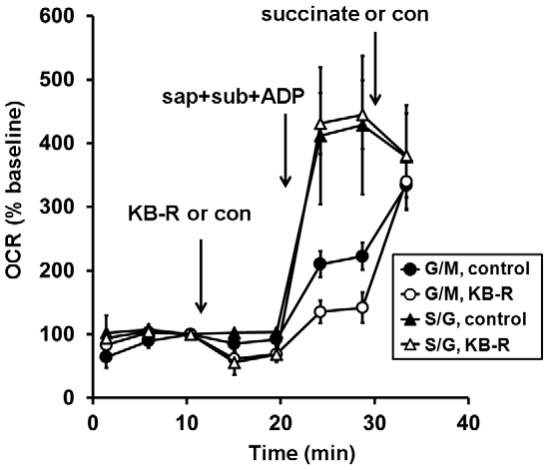
Inhibition of respiration by KB-R7943 is reversed by the complex II substrate succinate. Primary rat cortical neurons were control-treated (filled symbols) or treated with KB-7943 (K-BR, 30 µM, open symbols) after three baseline O_2_ consumption rate (OCR) measurements (first arrow). Neurons were then permeabilized by saponin (sap, 25 µg/ml) plus EGTA (5 mM) in aCSF medium containing glutamate/malate (G/M, 3 mM and 1 mM, respectively, circles) or succinate/glutamate (S/G, 3 mM each, triangles) as substrates (sub), ADP (1 mM), and excess K_2_PHO_4_ (3.6 mM for 4 mM final) to measure ADP-stimulated respiration (second arrow). Neurons permeabilized in the presence of G/M received a subsequent succinate (5 mM) addition (third arrow, circles) while neurons already exposed to succinate received a control (con) injection (triangles). OCRs are mean ± SD in triplicate, normalized to the third measurement point and expressed as % baseline OCR.

Brustovetsky et al. showed that KB-R7943 inhibited the respiration of isolated brain mitochondria oxidizing malate/glutamate but not of mitochondria oxidizing succinate/glutamate [10]. We treated neurons with KB-R7943 (30 µM) to inhibit respiration and subsequently permeabilized cells in the presence of ADP and malate/glutamate or succinate/glutamate at the concentrations described [10]. ADP-stimulated state 3 respiration was impaired in KB-R7943-treated permeabilized neurons in the presence of malate/glutamate but not in the presence of succinate/glutamate (Fig. 6). The subsequent addition of succinate to neurons oxidizing complex I substrates stimulated OCR to the level of the control, rescuing KB-R7943-mediated respiratory inhibition. These results suggest that the reduction of respiration by KB-R7943 in intact neurons is primarily due to the inhibition of complex I.

## Discussion

Measurement of O_2_ consumption using isolated brain mitochondria is the most widely used technique to study mitochondrial bioenergetic dysfunction in neurodegenerative disorders. The ability to supply mitochondria with defined combinations of substrates and inhibitors is a distinct advantage compared to intact cells. However, the use of tissue homogenization/density gradient centrifugation to isolate mitochondria is a disadvantage, as it may influence mitochondrial structure/function and likely selects for a subpopulation of mitochondria [28], [29]. Furthermore, isolated brain mitochondria are derived from both neurons and glia, precluding the study of mitochondria from individual cell populations. In this study we developed a microplate-based method using the Seahorse XF24 to measure respiration from permeabilized primary neurons. This technique allows for the exposure of mitochondria to defined substrate/inhibitor combinations without many of the disadvantages associated with mitochondrial isolation. In addition, we optimized assay conditions to for the first time allow consecutive respiration measurements from intact and subsequently permeabilized neurons.

A major benefit of our microplate-based method is that we used only 8×10^4^ cells per measurement, compared to a previous O_2_ electrode-based method which used 8.5×10^6^ neurons per measurement [7]. The small measurement volume (∼7 µl) within the XF24 plates enabled this large reduction in cell number which should make additional types of studies feasible, e.g. the evaluation of knockout/transgenic neurons prepared from single mouse embryos. In the present study, the microplate format allowed us to compare permeabilized and intact neurons on the same plate, as well as test a number of different substrate combinations in parallel.

We obtained excellent respiratory control ratios for mitochondria within permeabilized cortical neurons oxidizing either complex I or complex II substrates (Table 1). RCRs were similar or superior to most measurements made with isolated rat brain or cortical neuron mitochondria [18], [25]–[27]. State 4 respiration measured in the presence of oligomycin and complex I substrates was not significantly different compared to intact cells, suggesting that mitochondria in neurons permeabilized by saponin remained fully coupled. However, FCCP-stimulated respiration in intact cells was significantly higher that FCCP-stimulated respiration in permeabilized cells. The FCCP response in permeabilized neurons greatly improved if FCCP was added simultaneous to permeabilization rather than after oligomycin and a substantial time lag (Fig. 3B). Nevertheless, uncoupled respiration declined over time while ADP-stimulated respiration was relatively stable. In preliminary experiments, the FCCP response after oligomycin in permeabilized neurons was not improved by protease inhibitor cocktail (1∶500), the antioxidant Trolox (50 µM), or the mitochondrially-targeted antioxidant mito-TEMPO (10 or 50 µM, unpublished observations). The finding that FCCP-stimulated OCR was significantly higher when pyruvate/malate compared to glutamate/malate were used as substrates (Table 1) suggests that impaired FCCP OCRs may be due to defects in substrate transport. Testing this and other hypotheses will be the subject of future investigations.

In recent years the spare respiratory capacity of mitochondria has emerged as an important variable that influences neuronal survival in response to energetic or oxidative stress [30]. This parameter was defined as the difference between basal respiration and maximal respiration measured in the presence of uncoupler. However, respiration in the presence of FCCP is not limited by the ATP synthase and may overestimate the spare capacity of mitochondria that is available for ATP production. Using the acute saponin permeabilization technique developed in this study, it is possible to estimate the spare respiratory capacity by measuring the difference between basal and ADP-stimulated respiration. A slight overestimation of spare capacity when using FCCP was indeed suggested by the data in Fig. 3B where FCCP-stimulated OCR of intact neurons (open squares) was higher than ADP-stimulated OCR of permeabilized neurons (filled triangles) when measured in parallel at the same time point (injection *a*). The basal OCR of cultured neurons was much closer to the state 4 than the state 3 respiration rate, demonstrating that neurons at rest have a modest energy demand and substantial spare respiratory capacity.

It is worth noting that while our technique allows side-by-side or sequential comparisons of intact and permeabilized neurons, OCRs in permeabilized neurons may differ from those in intact cells due to substrate supply. We found that 80–90% of OCR in intact cortical neurons metabolizing glucose was rotenone-sensitive (Fig. 5A, filled squares, and data not shown), suggesting that it is reasonable to compare permeabilized neurons oxidizing the complex I substrates pyruvate/malate or glutamate/malate to intact cells. However, it will be interesting in the future to compare more complex substrate mixtures in permeabilized cells to intact cells, both in neurons and in other cells types.

We investigated the effects of the drug KB-R7943 on O_2_ consumption to illustrate how the acute saponin permeabilization technique can be used to address the mechanism of an electron transport deficit observed in cells. KB-R7943 inhibited basal respiration and markedly impaired spare respiratory capacity, estimated using either the FCCP OCR in intact neurons (Fig. 4) or the ADP complex I-linked OCR in permeabilized neurons (Fig. 6). Although recent experiments with isolated mitochondria suggested that KB-R7943 directly inhibits complex I [10], a number of other functions have been ascribed to KB-R7943, including inhibition of sodium-calcium exchange [11], inhibition of sodium and calcium permeable NMDA receptors [10], and inhibition of mitochondrial calcium uptake [31]. The multitude of targets raised the possibility that KB-R7943 also impacts mitochondrial function indirectly, e.g. via cellular sodium and calcium changes. However, we found that impaired respiratory capacity in intact neurons treated with KB-R7943 was completely reversed by the delivery of the complex II substrate succinate via acute permeabilization (Fig. 6). This finding suggests that inhibition of complex I-linked respiration is the major mechanism of KB-R7943-mediated respiratory inhibition in intact cells.

Surprisingly, methyl succinate did not relieve the KB-R7943-induced respiratory inhibition in neurons (Fig. 4B) despite previous evidence suggesting that it is cell permeable [24], [32]. One study found that methyl succinate could promote membrane potential repolarization of mitochondria within hippocampal neurons following transient excitotoxic glutamate exposure [24]. However, electron transport chain inhibitors were not added to confirm that the mechanism of repolarization was through complex II. In another study, methyl succinate stimulated insulin release from pancreatic islets in a concentration-dependent manner [32]. However, insulin secretion was blocked by the complex I inhibitor rotenone, suggesting that methyl succinate did not promote beta cell metabolism through complex II. Similar to this second study, we found that methyl succinate could not support neuronal respiration in the presence of rotenone (Fig. 5A). Methyl succinate was also a poor complex II substrate in permeabilized cells (Fig. 5B). The mechanisms by which methyl succinate influences the metabolism of intact cells require further investigation. Importantly, our studies demonstrate that although methyl succinate does not effectively promote complex II-linked respiration in neurons, delivery of succinate itself via acute plasma membrane permeabilization is a viable alternative to bypass complex I deficits.

In summary, we have developed a novel microplate-based method to measure respiration from permeabilized neurons. Our assay medium is compatible with intact or permeabilized cells, allowing us to treat intact cells and then acutely permeabilize to investigate respiratory chain function. We confirmed that saponin at the concentration used does not compromise mitochondrial outer membrane integrity by showing that exogenous cytochrome *c* fails to stimulate respiration (Fig. 2). Although here we report optimization of the method only for neurons, the method can be applied to additional cell types if careful cell density and saponin titrations are performed together with the cytochrome *c* test to confirm mitochondrial outer membrane integrity (unpublished observations). There is a lot of interest in finding new, higher throughput ways to study mitochondrial dysfunction. As life expectancy increases, the prevalence of neurodegenerative diseases is also increasing. The adaptation of microplate-based respirometry for permeabilized neurons should facilitate the evaluation of electron transport chain deficits in acute and chronic neurodegenerative disorders.
